# *Brevundimonas aurifodinae*, sp. nov., an Aerobic Anoxygenic Phototroph Resistant to Metalloid Oxyanions Isolated from Gold Mine Tailings

**DOI:** 10.3390/microorganisms12112167

**Published:** 2024-10-27

**Authors:** Chris Maltman, Katia Messner, John A. Kyndt, Vladimir Yurkov

**Affiliations:** 1 Department of Microbiology, University of Manitoba, Winnipeg, MB R3T 2N2, Canada; christopher.maltman@sru.edu (C.M.); messner1@myumanitoba.ca (K.M.); 2 Department of Biology, Slippery Rock University, Slippery Rock, PA 16057, USA; 3 College of Science and Technology, Bellevue University, Bellevue, NE 68005, USA; jkyndt@bellevue.edu

**Keywords:** aerobic anoxygenic phototroph, *Brevundimonas*, *Brevundimonas aurifodinae*, Bacteriochlorophyll *a*, mine tailings, metalloid oxide resistance, tellurium, selenium, vanadium

## Abstract

A polyphasic taxonomic study was carried out on the rod-shaped, orange-pigmented strain C11^T^, isolated from gold mine tailings. Sequencing of the 16S rRNA gene showed a relatedness to *Brevundimonas*, with a 98.4% and 98.2% similarity to *Brevundimonas bacteroides* and *Brevundimonas variabilis*, respectively. The average nucleotide identity and a digital DNA–DNA hybridization with the closest phylogenetic neighbor of strain C11^T^ indicate distinction at the species level, further confirmed by the differences in physiology. C_18:1_ ω7c is the dominant cellular fatty acid. Its DNA G + C content is 68.3 mol %. Its predominant ubiquinone is Q-10; 1,2-Di-O-acyl-3-O-α-D-glucopyranuronosyl glycerol, phosphatidylglycerol, 1,2-di-O-acyl-3-O-α-D-glucopyranosyl glycerol, and 1,2-di-O-acyl-3-O-[D-glucopyranosyl-(1→4)-α-D-glucopyranuronosyl] glycerol are its major polar lipid constituents. This bacterium produces bacteriochlorophyll *a* and tolerates high concentrations of (μg/mL) the following: tellurium (>1500), selenium (1000 to >5000), and vanadium (>5000) oxyanions. The data support the inclusion of the strain C11^T^ into the genus *Brevundimonas* as a new species with the proposed name *Brevundimonas aurifodinae* sp. nov. (C11^T^ = NRRL B-65718^T^; =DSM 118059^T^).

## 1. Introduction

*Brevundimonas* is a genus, belonging to the family *Caulobacteraceae*, that was proposed in 1994 to re-classify two species, which had a distinct taxonomic position among the *Pseudomonas* [1]. It is also closely related to *Caulobacter*, with several current *Brevundimonas* members formerly residing in the genus [2]. Currently, there are 37 validly recognized members with *B. diminuta* as their type species [3]. The members are gram-negative, rod-shaped, aerobic, oligotrophic, contain Q-10 as the major isoprenoid quinone, and have a relatively high DNA G + C content [4]. However, they vary in traits such as motility, pigmentation, and prosthecae formation [4]. Species have been isolated from a wide range of habitats including the following: soils, marine/freshwater environments, sand, and activated sludge [5,6]. *Brevundimonas* spp. are becoming of greater interest as human pathogens, with *B. diminuta* and *B. vesicularis* being found in clinical specimens of patients with underlying conditions [6]. One interesting feature is that some members produce bacteriochlorophyll *a* (Bchl *a*), and contain all the genes necessary for anoxygenic photosynthesis, indicating they can be classified as aerobic anoxygenic phototrophs (AAPs) [7]. Currently, three species are known to have this ability: *B. bacteroides* [7], *B. subvibrioides* [8], and *B. variabilis* [9]. AAPs are a diverse group found in many different environments and various genera of the *Proteobacteria* [10]. Their core characteristic is the ability to supplement their obligately aerobic, heterotrophic metabolism with energy derived from Bchl *a*-based phototrophy [11]. A standout characteristic of AAPs is their ability to withstand extremely high levels of metalloid oxyanions [10]. Resistance to toxic metals is a known attribute of many *Brevundimonas* spp. For example, *B. vancanneytii* SMA3 mitigates the heavy metals cadmium, lead, and mercury from soils [12], *B. diminuta* can survive increased levels of arsenic, cadmium, and zinc [13,14], while *B. vesicularis* remediates copper, lead, and nickel [15,16]. Previously, the tailings of the Central Gold Mine in Nopiming Provincial Park, in Manitoba, Canada were investigated to explore the diversity of AAPs in this extreme environment and their resistance to metalloid oxides [17]. From here, the strain C11^T^ was isolated and found to produce Bchl *a*, resist high levels of Te, Se, and V oxyanions as well as have close relatedness to *Brevundimonas* [17]. Since the genus has potential for bioremediation, the discovery of more species which tolerate and remove toxic metals could lead to future applications. As such, we set forth to taxonomically classify strain C11^T^ as a new species with the proposed name *Brevundimonas aurifodinae*.

## 2. Materials and Methods

### 2.1. Strains and Cultivation

Strain C11^T^ was isolated from gold mine tailings at Nopiming Provincial Park, in Manitoba, Canada using a Rich Organic (RO) solid medium [17]. The cells in all experiments were grown aerobically on a *Caulobacter* medium [18] at 30 °C and pH 7.0 in the dark unless otherwise noted. Long term storage was at −80 °C in 30% glycerol that was RO-modified to contain 10% (*w*/*v*) of the original organic content. The following strains were obtained for comparison: from the Korean Agricultural Culture Collection: *Brevundimonas alba* KACC 12015^T^ [2], *Brevundimonas bacteroides* KACC 12013^T^ [2], *Brevundimonas basaltis* KACC 17487^T^ [19], *Brevundimonas subvibrioides* KACC 12014^T^ [2], and *Brevundimonas variabilis* KACC 12016^T^ [2]; and from the USDA-ARS Culture Collection (NRRL): *Brevundimonas diminuta* NRRL B-1496^T^ [1].

### 2.2. Physiological and Biochemical Experiments

Taxonomical markers such as the production of specific enzymes and utilization of carbon sources were investigated using API ZYM and API 20NE test strips (Biomeriux, Durham, NC, USA) and a Biolog GEN III Microplate (Biolog Inc., Hayward, CA, USA). Motility was determined by the hanging drop method [20]. In addition to the antibiotic susceptibility tested with the Biolog GEN III Microplate, disk diffusion assays were carried out using the BD BBL™ Sensi-Disc™ [21] with the following: penicillin G (10 IU), ampicillin (10 µg), polymyxin B (300 IU), tetracycline (30 µg), erythromycin (15 µg), imipenem (10 µg), streptomycin (10 µg), chloramphenicol (30 µg), bacitracin (2 IU), kanamycin (30 µg), and rifampin (5 µg). Temperature range for growth was evaluated from 0 to 50 °C at 5 °C intervals while pH tolerance was assessed from 4.0 to 11.0 with 0.5 increments and NaCl from 0.0 % to 6.0 % at 0.5 % intervals. Gram stain, spore formation, anaerobic growth, catalase, oxidase, methyl red, Voges–Proskauer, indole, and other carbon source utilization tests were completed as described [22].

Anoxygenic photosynthetic complex formation and Bchl *a* synthesis were assessed with the absorption spectra taken from whole cells and pigment extracts using the standard methods with the Hitachi U-2010 spectrophotometer after the cells were grown for 7 days on RO plates [11]. Aerobic photoautotrophic growth; aerobic and anaerobic nitrate, ammonia and N_2_, utilization; as well as the resistance and reduction of metal(loid) oxides were evaluated previously [17].

For fatty acid profiling, strain C11^T^ was grown in a *Caulobacter* medium at 30 °C for 72 h. Biomass was collected; lipids were extracted with the Folch method [23] and analyzed via gas chromatography [24]. Polar lipids were discerned using two-dimensional thin-layer chromatography with the appropriate detection reagents [25]. Cellular quinones obtained from 100 mg of freeze-dried cells were separated by TLC and identified [25].

### 2.3. Microscopy

The cell size and shape of a 48 h culture was observed with a phase contrast light microscope (Zeiss Axioskop 2, Carl Zeiss AG, Oberkochen, Germany).

### 2.4. Phylogenetic Analysis

DNA was extracted as in protocol [26] and sent to Azenta (South Plainfield, NJ, USA) for 16S rRNA gene Sanger sequencing using the universal primers 27F (5′-AGAGTTTGATCCTGGCTCAG-3′) and 1492R (5′-GGTTACCTTGTTACGACTT-3′). A 1404 bp fragment (GenBank accession number: PP885399) was produced. The closest relatives were identified with a NCBI standard nucleotide BLAST search. With the pairwise aligned 16S rRNA fragments of other *Brevundimomas* spp. collected from the NCBI GenBank, a Maximum Likelihood phylogenetic tree was created in MEGA 11 [27]. The genome sequence of strain C11^T^ was obtained using a previously described approach [28]. Briefly, the sequencing library of the genomic DNA was prepared using the Illumina DNA Library Prep kit. The genome was sequenced with the Illumina MiniSeq platform using 500 μL of a 1.8 pM library. Paired-end (2 × 150 bp) sequencing generated 2,690,380 reads and 406.3 Mbps. Quality control of the reads was performed using FASTQC (v1.0.0), using a k-mer size of 5 and contamination filtering for overrepresented sequences against the default contamination list. Genome assembly with the Illumina sequencing was performed using Unicycler (v0.5.0) [29] within BV-BRC [30]. This resulted in a 3.3 Mbp genome consisting of 26 contigs (116× coverage). It was then annotated using the NCBI prokaryotic genome annotation pipeline [31]. The Whole Genome Shotgun project has been deposited at the DDBJ/ENA/GenBank under the accession JBEGDD000000000. The version described in this paper is version JBEGDD010000000. The average nucleotide identity (ANI) was assessed with the OrthoANI algorithm through ChunLab’s online ANI calculator [32]. Formula d_4_ of the Genome–Genome Distance Calculator from DSMZ [33] was used to define the digital DNA–DNA hybridization (dDDH) values. A Genome Blast Distance Phylogeny tree was created with the Type Strain Genome Server (TYGS) from DSMZ [34]. Intergenomic distances were applied to generate a balanced minimum evolution tree via FASTME 2.1.6.1 including SPR post-processing [35]. Branch support was inferred from 100 pseudo-bootstrap replicates for each. The genome-based tree was rooted at the midpoint [36] and visualized with PhyD3 [37].

## 3. Results and Discussion

### 3.1. Physiology and Morphology of Cells

**Figure 1:**
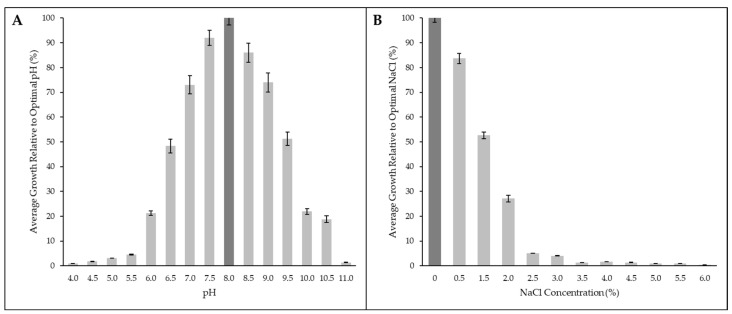
Strain C11^T^ growth at different pH (**A**) and NaCl concentrations (**B**). The optimum is the darkened bar. Standard deviations are added.

The API ZYM and API 20NE test strips, and the Biolog Gen III Microplate revealed that strain C11^T^ used a variety of carbon sources for growth and confirmed the activity of a set of enzymes which are outlined in the species description (Section 4.1). The carbon sources and enzyme activities not cited here which were included in these test kits, produced negative results. Strain C11^T^ does not require vitamin supplements. Growth occurs between 5 and 40 °C, from a pH of 6.0 to 10.5, and up to 2.0 % NaCl with the optima at 30 °C, pH 8.0, and 0 % NaCl (Table 1, Figure 1). Strain C11^T^ could neither fix nitrogen gas nor reduce nitrates, only using ammonia as an inorganic nitrogen source aerobically. However, organic nitrogen sources both simple (amino acids) and complex (casamino acids, bactopeptone, yeast extract) are utilized for growth by strain C11^T^ in oxygenated conditions (a full list of the carbon sources used, including the nitrogen-containing compounds, is in the species description, Section 4.1). It is incapable of growing photoautotrophically.

Strain C11^T^ is susceptible to chloramphenicol, kanamycin, polymyxin B, streptomycin, imipenem, vancomycin, tetrazolium violet, tetrazolium blue, aztreonam, macrolide, rifamycin, minocycline, lincomycin, guanidine HCl, niaproof 4, fusidic acid, D-serine, and sodium bromate, but resistant to nalidixic acid, lithium chloride, penicillin, and ampicillin.

Strain C11^T^ was assessed for its ability to resist and potentially reduce metal(loid) oxyanions [17]. It had the broadest and greatest tolerance among the other isolates from the gold mine tailings in Nopiming Provincial Park. This included surviving high levels of (µg/mL) the following: tellurite (>1500), tellurate (>1500), selenite (1000), selenate (>5000), metavanadate (>5000), and orthovanadate (>5000). Furthermore, the strain C11^T^ has potential bioremediation and biometallurgy applications as it reduced tellurite to elemental tellurium [17].

**Figure 2:**
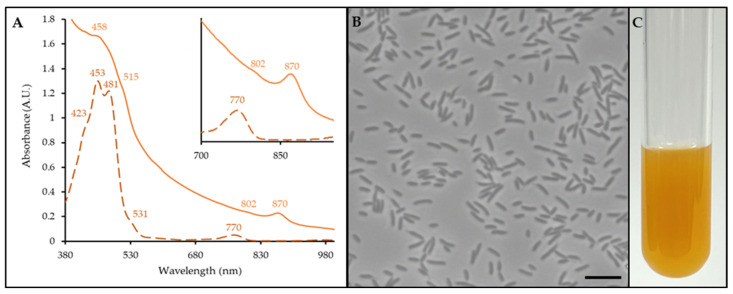
Photosynthetic complex, cell morphology, and pigmentation of strain C11^T^. (**A**) Whole cell (light orange line) and pigment extract (dark orange, dashed line) absorbance spectra. Peaks and shoulders of importance are indicated. (**B**) Phase contrast micrograph of cells. Bar is 5 μm. (**C**) Liquid culture.

In vivo, it has an anoxygenic photosynthesis complex containing a light-harvesting I complex (870 nm) and a reaction center (802 nm) (Figure 2A, light orange line). Bchl *a* was detected in the pigment extract absorbance spectrum (770 nm, Figure 2A, dashed dark orange line). As such, strain C11^T^ is classified as an aerobic anoxygenic phototroph. The carotenoids were also synthesized (Figure 2A, orange line: 458, 515 nm; Figure 2A, dashed dark orange line: 423, 453, 481, and 531 nm).

Strain C11^T^ is gram-negative, oxidase- and catalase-positive, non-spore forming, and obligately aerobic. It produces circular (1–2 mm), raised, orange colonies with entire margins and a mucoid consistency on *Caulobacter* media plates after 72 h. Morphologically, the cells are rod shaped, 1.5–2.0 µm in length, 0.75–1.0 µm in width, and do not form prosthecae after 48 h of growth (Figure 1B).

### 3.2. Chemotaxonomic Characterization

The whole cell fatty acid analysis revealed strain C11^T^ contains predominately C_18:1_ ω7c. The major polar lipids are 1,2-di-O-acyl-3-O-α-D-glucopyranuronosyl glycerol (MGDOx), phosphatidylglycerol (PG), 1,2-di-O-acyl-3-O-α-D-glucopyranosyl glycerol (MGD), and 1,2-di-O-acyl-3-O-[D-glucopyranosyl-(1→4)-α-D-glucopyranuronosyl]glycerol (DGL). MGDOx production differentiates *Brevundimonas* from *Caulobacter* and, as such, its presence in the strain C11^T^ supports the conclusions of this study [4]. The predominant ubiquinone in the cells is Q-10. The fatty acid, polar lipid, and quinone profiles are representative of the *Brevundimonas* spp. [4], solidifying its genus placement.

### 3.3. Phylogenetic and Genomic Analysis

**Figure 3:**
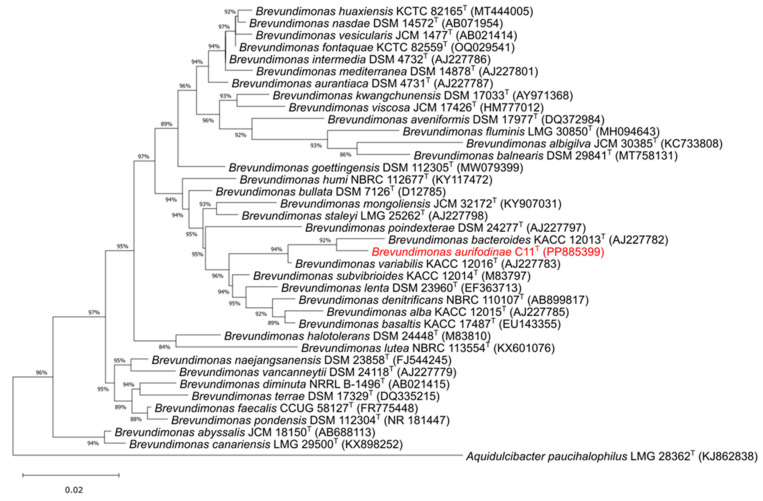
Maximum Likelihood 16S rRNA phylogenetic tree of strain C11^T^ and validated species in *Brevundimonas*. The scale bar represents the amount of substitutions per nucleotide. Accession numbers are in parenthesis. Value at nodes indicate the bootstrap support calculated with a neighbor joining analysis of 1000 resampled datasets; only values > 50% are shown. *Aquidulcibacter paucihalophilus* LMG 28362^T^ was used as the outgroup.

A nearly complete 16S rRNA fragment (1404 bp) of strain C11^T^ was produced via Sanger sequencing with the full gene (1461 bp) identified in the genome on contig 7. Pairwise comparisons to the type species revealed a 16S rRNA relatedness of 98.4 % to *B. bacteroides* KACC 12013^T^ [2] and 98.2 % to *B. variabilis* KACC 12016^T^ [2]. Using these sequences and those from the validated *Brevundimonas* members, a Maximum Likelihood 16S rRNA phylogenetic tree was created (Figure 3). The final product positions strain C11^T^ within the genus (Figure 3).

**Figure 4:**
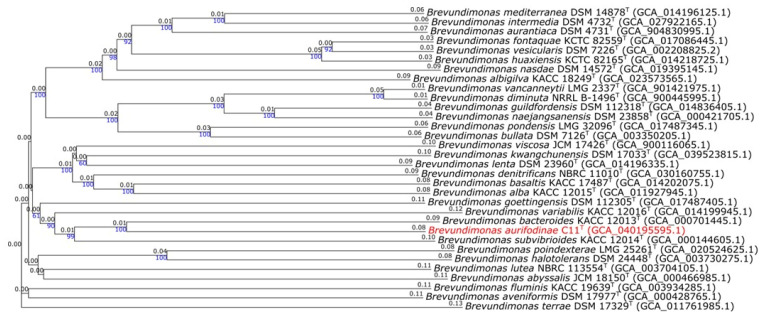
Genome Blast Distance Phylogeny tree of strain C11^T^ among species of the genus *Brevundimonas*. The blue numbers on branches are the pseudo-bootstrap support values > 60% from 100 replications, with an average branch support of 81.3%. The black numbers represent the branch length. The tree was rooted at the midpoint.

The genome of strain C11^T^ is 3.3 Mb and it has a G + C content of 68.3 mol %, falling within the range of the other genus members (Table 2) [4]. It contains 26 contigs, has an L50 of 5, and 3186 protein-coding genes were annotated. The OrthoANI between strain C11^T^ and *B. bacteroides* is 83.5% and the dDDH is 26.6 %. For *B. variabilis*, it is 77.7 % and 21.8 %, respectively. All the values fall below the accepted cutoffs for species differentiation [38]. The Genome Blast Distance Phylogeny tree created shows a distinct lineage for strain C11^T^ among the other *Brevundimonas* spp. that supports its classification as a new species (Figure 4).

While the cells were non-motile under the test conditions, strain C11^T^ possesses many of the genes for flagellum production as well as a chemotaxis regulator *cheY*. However, the gene *filC*, which encodes the flagellum filament, is absent from the genome, explaining the non-motile phenotype observed [39]. As shown via spectrophotometry, it synthesizes Bchl *a* and as such, has all the necessary sequences. These genes alongside the others required for photosynthesis (such as light-harvesting complex and reaction center production) are found within a single cluster in the chromosome. Furthermore, the genes encoding RuBisCo are not found in the genome. Alternatively, a gene encoding phosphoenolpyruvate carboxylase is present, suggesting strain C11^T^ conducts anaplerotic carbon fixing, an activity known to take place in AAPs which is insufficient for autotrophic growth [10]. No nitrogen fixation genes or nitrate reductase was found, supporting the results described in Section 3.1. Besides being resistant to very high levels of tellurite, tellurate, selenite, selenate, metavanadate, and orthovanadate [17], copper and silver resistance via a resistance-nodulation-division efflux transporter is also encoded, suggesting strain C11^T^ may be able to tolerate high levels of Cu and Ag. These genes alongside those encoding for conjugative transfer, the enzyme polygalacturonase, as well as additional hypothetical proteins, comprise the 404 PATRIC cross-genus families found in strain C11^T^ but not in its closest relatives *B. bacteroides*, *B. subvibrioides*, and *B. variabilis*. This supports the evidence provided that there is sufficient variation in the features of strain C11^T^ which distinguishes it from other *Brevundimonas* spp.

## 4. Conclusions

The strain C11^T^, isolated from gold mine tailings in Nopiming Provincial Park, possesses significant differences in both phenotype (carbon metabolism and enzyme activities) and genotype (ANI and dDDH), which sufficiently differentiate it from its closest relatives. As such, this bacterium represents a new species in the genus and the name *Brevundimonas aurifodinae* is proposed.

### 4.1. Description of Brevundimonas aurifodinae sp. nov.

*Brevundimonas aurifodinae* (au.ri.fo.di’nae. L. neut. n. aurum, gold; L. fem. n. fodina, mine; N.L. gen. n. aurifodinae, indicates discovery at a gold mine) is Gram-negative, non-motile, non-spore forming, and obligately aerobic. Circular (1–2 mm), raised, orange colonies with entire margins and a mucoid consistency formed on the *Caulobacter* medium plates after 72 h. The cells are rod-shaped, 1.5–2.0 μm in length and 0.8–1.0 μm in width, non-prosthecate, and catalase- and oxidase-positive. Growth occurs in the following conditions (optimum): between 5 and 40 °C (30 °C), from a pH of 6.0 to 10.5 (8.0), and up to 2.0% NaCl (0%). The carbon sources utilized include the following: Gluconate, dextrin, D-cellobiose, gentibiose, sucrose, D-turanose, stachyose, α-D-lactose, D-melibiose, N-acetyl-D-glucosamine, N-acetyl-beta-D-mannosamine, N-acetyl-D-galactosamine, N-acetyl-muraminic acid, α-D-glucose, D-mannose, D-galactose, 3-methyl-glucose, D-fucose, L-fucose, L-rhamnose, D-mannitol, D-arabitol, myo-inositol, D-glucose-6-phosphate, D-fructose-6-phosphate, D-aspartic acid, glycyl-L-proline, L-alanine, L-aspartic acid, L-glutamic acid, L-serine, D-galacuronic acid, L-galacturonic acid, D-gluconic acid, D-glucuronic acid, glucuronamide, quinic acid, D-saccharic acid, D-lactic acid methyl ester, citric acid, α-keto-glutaric acid, D-malic acid, L-malic acid, bromosuccinic acid, α-hydroxybutyric acid, β-hydroxy-D, L-butyric acid, α-keto-butyric acid, acetoacetic acid, acetic acid, casamino acids, yeast extract, and bactopeptone. Alternatively, capric acid, adipic acid, phenylacetic acid, D-maltose, D-trehalose, D-raffinose, β-methyl-D-glucoside, D-salicin, D-fructose, inosine, D-sorbitol, glycerol, D-serine, L-arginine, L-histidine, L-pyroglutamic acid, pectin, mucic acid, p-hydroxy-phenylacetic acid, methyl pyruvate, L-lactic acid, ɣ-amino-butyric acid, propionic acid, ethanol, methanol, and formic acid were not used. Can grow without vitamin supplements. Alkaline phosphatase, esterase (C4), esterase lipase (C8), leucine arylamidase, valine arylamidase, trypsin, α-chymotrypsin, acid phosphatase, naphthol-AS-bi-phosphohydrolase, α-glucosidase, and amylase activities were present, while arginine dihydrolase, lipase, cystine arylamidase, α-galactosidase, β-galactosidase, β-glucuronidase, β-glucosidase, N-acetyl-β-glucosaminidase, α-mannosidase, α-fucosidase, urease, and nitrate reductase were not. Esculin and gelatin were hydrolyzed. The indole, methyl red, and Voges–Proskauer tests were negative. The primary fatty acid was C_18:1_ ω7c. Ubiquinone Q-10 was the dominant isoprenoid quinone. MGDOx, PG, MGD, and DGL were the major polar lipids. It produces bacteriochlorophyll *a* and is resistant to high levels of (μg/mL) the following: tellurite (>1500), tellurate (>1500), selenite (1000), selenate (>5000), metavanadate (>5000), and orthovanadate (>5000). It can reduce tellurite to elemental tellurium. The DNA G + C content is 68.3 mol %.

The type strain C11^T^ (=NRRL B-65718^T^ = DSM 118059^T^) was isolated from gold mine tailings at Nopiming Provincial Park, in Manitoba, Canada. The strain C11^T^ ribosomal 16S rRNA gene sequence is available under the GenBank accession number: PP885399. This Whole Genome Shotgun project has been deposited at the DDBJ/ENA/GenBank under the accession JBEGDD000000000, which was used in this study.

## Figures and Tables

**Table 1:** Physiological and biochemical features of strain C11^T^ compared to related members and the type species of the genus *Brevundimonas* ^1,2^.

Species	*B. aurifodinae*	*B. bacteroides*	*B. variabilis*	*B. basaltis*	*B. alba*	*B. subvibrioides*	*B. diminuta*
Strain	C11^T^	KACC 12013^T^	KACC 12016^T^	KACC 17487^T^	KACC 12015^T^	KACC 12014^T^	NRRL B-1496^T^
Temperature (°C)	5–40	25–40	10–40	10–40	10–40	25–40	10–40
Optimum	30	30	30	30	30	30	30
pH	6.0–10.5	6.0–8.0	6.0–8.0	5.5–10.0	6.0–8.0	6.0–8.0	6.0–8.0
Optimum	8.0	7.0	7.0	7.5	7.0	7.0	7.0
NaCl Tolerance (%)	2	4	4	4	2	2	6
Optimum	0	0	0	0	0	0	0
Utilization of:							
D-maltose	−	−	+	−	−	−	+
D-cellobiose	+	−	+	−	−	+	+
Gentibiose	+	+	+	+	−	−	+
Sucrose	+	−	+	−	−	−	+
Stachyose	+	+	+	−	−	−	+
D-raffinose	−	−	+	−	−	+	+
β-methyl-D-glucoside	−	+	−	+	−	+	+
D-salicin	−	+	+	−	−	+	+
α-D-glucose	+	−	+	+	+	+	+
D-mannose	+	+	−	−	+	+	+
D-fructose	−	−	−	+	−	−	+
Gelatin	+	+	+	−	+	−	+
L-arginine	−	+	+	+	−	−	+
Pectin	−	−	+	+	−	+	+
Mucic acid	−	+	+	+	−	+	+
D-malic acid	+	+	−	+	−	+	+
α-hydroxy-butyric acid	+	+	−	−	−	−	+
α-keto-butyric acid	+	−	−	−	−	−	+
Propionic acid	−	+	−	+	−	−	−
Enzyme Activities:							
Amylase	+	+	+	−	−	+	−
Gelatinase	+	+	+	−	+	−	−
Leucine arylamidase	+	−	+	+	+	+	+
Valine arylamidase	+	−	+	+	+	+	+
Cysteine arylamidase	−	−	−	+	−	+	−
α-chymotrypsin	+	−	+	+	+	+	+
α-glucosidase	+	+	+	+	+	+	−
Oxidase	+	+	−	−	+	+	+
Esculin Hydrolysis	+	+	+	+	+	+	−

^1^+, Growth occurs, enzyme activity detected; −, No growth, no enzyme activity; ^2^ Physiological results not included here produced identical results among those tested.

**Table 2:** General genome features of strain C11^T^ compared to related members, and the type species of *Brevundimonas.*

Species	*B. aurifodinae*	*B. bacteroides*	*B. variabilis*	*B. basaltis*	*B. alba*	*B. subvibrioides*	*B. diminuta*
Strain ^1^	C11^T^	KACC 12013^T^	KACC 12016^T^	KACC 17487^T^	KACC 12015^T^	KACC 12014^T^	NRRL B-1496^T^
16S rRNA Gene Similarity (%) ^2^	100	98.4	98.2	97.5	97.4	97.2	94.8
Genome Size (Mb)	3.3	3.2	3.4	2.6	3.1	3.4	3.4
G + C Content	68.3	68.2	65.3	68.5	68.6	68.4	67.3
Genes	3258	3225	3320	2715	3056	3385	3438
Protein-coding genes	3186	3169	3246	2649	3000	3325	3358
No. of contigs	26	17	8	8	3	1	2
No. of tRNA genes	43	44	46	43	46	47	53
L50	5	4	2	2	1	1	1
OrthoANI (%) ^2^	100	83.5	77.7	77.7	77.4	80.9	77.3
dDDH (%) ^2^	100	26.6	21.8	21.4	21.3	23.5	20.8

^1^ GenBank Assembly Accession numbers of strains from left to right: GCA_040195595.1, GCA_000701445.1, GCA_014199945.1, GCA_014202075.1, GCA_011927945.1, GCA_000144605.1, and GCA_900445995.1. ^2^ Compared to strain C11^T.^

## Data Availability

Strain C11^T^ ribosomal 16S rRNA gene sequence is available under the GenBank accession number: PP885399. This Whole Genome Shotgun project has been deposited at the DDBJ/ENA/GenBank under the accession JBEGDD000000000, which was used in this study.

## References

[1] Segers P., Vancanneyt M., Pot B., Torck U., Hoste B., Dewettinck D., Falsen E., Kersters K., De Vos P. (1994). Classification of *Pseudomonas diminuta* Leifson and Hugh 1954 and *Pseudomonas vesicularis* Büsing, Döll, and Freytag 1953 in *Brevundimonas* gen. nov. as *Brevundimonas diminuta* comb. Nov. and *Brevundimonas vesicularis* comb. Nov., Respectively. Int. J. Syst. Evol. Microbiol..

[2] Abraham W.-R., Strompl C., Meyer H., Lindholst S., Moore E., Christ R., Vancanneyt M., Tindall B., Bennasar A., Smit J. (1999). Phylogeny and Polyphasic Taxonomy of *Caulobacter* species. Proposal of *Maricaulis* gen. nov. with *Maricaulis maris* (Poindexter) comb. nov. as the Type Species, and Emended Description of the Genera *Brevundimonas* and *Caulobacter*. Int. J. Syst. Evol. Microbiol..

[3] Parte A., Sardà Carbasse J., Meier-Kolthoff J., Reimer L., Göker M. (2020). List of Prokaryotic Names with Standing in Nomenclature (LPSN) moves to the DSMZ. Int. J. Syst. Bacteriol..

[4] Vancanneyt M., Segers P., Abraham W.-R., Vos P., Trujillo M.E., Dedysh S., DeVos P., Hedlund B., Kämpfer P., Rainey F.A., Whitman W.B. (2005). Brevundimonas. Bergey’s Manual of Systematics of Archaea and Bacteria.

[5] Friedrich I., Klassen A., Neubauer H., Schneider D., Hertel R., Daniel R. (2021). Living in a Puddle of Mud: Isolation and Characterization of Two Novel *Caulobacteraceae* Strains *Brevundimonas ondensis* sp. nov. and *Brevundimonas gottingensis* sp. nov. Appl. Microbiol..

[6] Ryan M., Pembroke J. (2018). *Brevundimonas* spp.: Emerging Global Opportunistic Pathogens. Virulence.

[7] Tanabe Y., Yamaguchi H., Yoshida M., Kai A., Okazaki Y. (2023). Characterization of a Bloom-Associated alphaproteobacterial Lineage, ‘*Candidatus* Phycosocius’: Insights into Freshwater Algal-Bacterial Interaction. ISME Comm..

[8] Imhoff J., Than T., Kunzel S., Neulinger S. (2019). Phylogeny of Anoxygenic Photosynthesis Based on Sequences of Photosynthetic Reaction Center Proteins and a Key Enzyme in Bacteriochlorophyll Biosynthesis, the Chlorophyllide Reductase. Microorganisms.

[9] Tahon G., Willems A. (2017). Isolation and Characterization of Aerobic Anoxygenic Phototrophs from Exposed Soils for the Sor Rondane Mountains, East Antarctica. Syst. Appl. Microbiol..

[10] Yurkov V., Hughes E., Hallenbeck P. (2017). Aerobic Anoxygenic Phototrophs: Four Decades of Mystery. Modern Topics in the Phototrophic Prokaryotes: Environmental and Applied Aspects.

[11] Yurkov V., Beatty T. (1998). Aerobic Anoxygenic Phototrophic Bacteria. Microbiol. Mol. Biol. Rev..

[12] Ghosh A., Sah D., Chakraborty M., Rai J. (2023). Bio-mediated Detoxification of Heavy Metal Contaminated Soils and Phytotoxicity Reduction Using Novel Strain of *Brevundimonas vancanneytii* SMA3. Heliyon.

[13] Singh N., Marwa N., Mishra S., Mishra J., Verma P., Rathaur S., Singh N. (2016). *Brevundimonas diminuta* Mediated Alleviation of Arsenic Toxicity and Plant Growth Promotion in *Oryza sativa* L.. Ecotoxicol. Environ. Saf..

[14] Ali A., Li M., Su J., Li Y., Wang Z., Bai Y., Ali E., Shaheen S. (2022). *Brevundimonas diminuta* Isolated from Mines Polluted Soil Immobilized Cadmium (Cd^2+^) and Zinc (Zn^2+^) Through Calcium Carbonate Precipitation: Microscopic and Spectroscopic Investigations. Sci. Total Environ..

[15] Resmi G., Thampi S., Chandrakaran S. (2010). *Brevundimonas vesicularis*: A Novel Bio-sorbent for Removal of Lead from Wastewater. Int. J. Environ. Res..

[16] Singh N., Gadi R. (2012). Bioremediation of Ni(II) and Cu(II) from Wastewater by the Nonliving Biomass of *Brevundimonas vesicularis*. J. Environ. Chem. Ecotoxicol..

[17] Hughes E., Head B., Maltman C., Piercey-Normore M., Yurkov V. (2017). Aerobic Anoxygenic Phototrophs in Gold Mine Tailings in Nopiming Provincial Park, Manitoba, Canada. Can. J. Microbiol..

[18] Poindexter J. (1964). Biological Properties and Classification of the *Caulobacter* Group. Bacteriol. Rev..

[19] Choi J.-H., Kim M.-S., Roh S., Bae J.-W. (2010). *Brevundimonas basaltis* sp. nov., Isolated from Black Sand. Int. J. Syst. Evol. Microbiol..

[20] Jordan E., Caldwell M., Reiter D. (1934). Bacterial Motility. J. Bacteriol..

[21] Bauer A., Kirby W., Sherry J., Turck M. (1966). Antibiotic Susceptibility Testing by Using a Standardized Single Disc Method. Am. J. Clin. Pathol..

[22] Holt J., Krieg N., Sneath P. (1994). Bergey’s Manual of Determinative Bacterology.

[23] Folch J., Lees M., Sloane-Stanley G.H. (1957). A Simple Method for the Isolation and Purification of Total Lipids from Animal Tissues. J. Biol. Chem..

[24] Estrela A., Abraham W. (2010). *Brevundimonas vancanneytii* sp. nov., Isolated from Blood of a Patient with Endocarditis. Int. J. Syst. Evol. Microbiol..

[25] Minnikin D.E., O’Donnell A.G., Goodfellow M., Alderson G., Athalye M., Schaal A., Parlett J.H. (1984). An Integrated Procedure for the Extraction of Bacterial Isoprenoid Quinones and Polar Lipids. J. Microbiol. Methods.

[26] Chen W., Kuo T. (1993). A Simple and Rapid Method for the Preparation of Gram-negative Bacterial Genomic DNA. Nucleic Acids Res..

[27] Tamura K., Stecher G., Kumar S. (2021). MEGA11: Molecular Evolutionary Genetics Analysis Version 11. Mol. Biol. Evol..

[28] Maltman C., Kuzyk S.B., Kyndt J., Lengyel G., Yurkov V. (2023). *Shewanella metallivivens* sp. nov., a Deep-sea Hydrothermal Vent Tube Worm Endobiont Capable of Dissimilatory Anaerobic Metalloid Oxyanion Reduction. Int. J. Sys. Evol. Microbiol..

[29] Wick R.R., Judd L.M., Gorrie C.L., Holt K.E. (2017). Unicycler: Resolving Bacterial Genome Assemblies from Short and Long Sequencing Reads. PLoS Comput. Biol..

[30] Wattam A.R., Davis J.J., Assaf R., Boisvert S., Brettin T., Bun C., Conrad N., Dietrich E.M., Disz T., Gabbard J.L. (2017). Improvements to PATRIC, the All-Bacterial Bioinformatics Database and Analysis Resource Center. Nucleic Acids Res..

[31] Tatusova T., DiCuccio M., Badretdin A., Chetvernin V., Nawrocki E.P., Zaslavsky L., Lomsadze A., Pruitt K.D., Borodovsky M., Ostell J. (2016). NCBI Prokaryotic Genome Annotation Pipeline. Nucleic Acids Res..

[32] Yoon S.H., Ha S.M., Lim J.M., Kwon S.J., Chun J. (2017). A Large-scale Evaluation of Algorithms to Calculate Average Nucleotide Identity. Antonie Leeuwenhoek.

[33] Meier-Kolthoff J.P., Sardà Carbasse J., Peinado-Olarte R.L., Göker M. (2021). TYGS and LPSN: A Database Tandem for Fast and Reliable Genome-based Classification and Nomenclature of Prokaryotes. Nucleic Acid Res..

[34] Meier-Kolthoff J., Goker M. (2019). TYGS is an Automated High-throughput Platform for State-of-the-art Genome-based Taxonomy. Nat. Comm..

[35] Lefort V., Desper R., Gascuel O. (2015). FastME 2.0: A Comprehensive, Accurate, and Fast Distance-based Phylogeny Inference program. Mol. Biol. Evol..

[36] Farris J.S. (1972). Estimating Phylogenetic Trees from Distance Matrices. Am. Nat..

[37] Kreft L., Botzki A., Coppens F., Vandepoele K., Van Bel M. (2017). PhyD3: A Phylogenetic Tree Viewer with Extended phyloXML Support for Functional Genomics Data Visualization. Bioinformatics.

[38] Richter M., Rossello-Mora R. (2009). Shifting the Genomic Gold Standard for the Prokaryotic Species Definition. Proc. Natl. Acad. Sci. USA.

[39] Liu R., Ochman H. (2007). Stepwise Formation of the Bacterial Flagellar System. Proc. Natl. Acad. Sci. USA.

