# Correction: Maltman et al. *Brevundimonas aurifodinae*, sp. nov., an Aerobic Anoxygenic Phototroph Resistant to Metalloid Oxyanions Isolated from Gold Mine Tailings. *Microorganisms* 2024, *12*, 2167

**DOI:** 10.3390/microorganisms14010086

**Published:** 2025-12-31

**Authors:** Chris Maltman, Katia Messner, John A. Kyndt, Vladimir Yurkov

**Affiliations:** 1 Department of Microbiology, University of Manitoba, Winnipeg, MB R3T 2N2, Canada; christopher.maltman@sru.edu (C.M.); messner1@myumanitoba.ca (K.M.); 2 Department of Biology, Slippery Rock University, Slippery Rock, PA 16057, USA; 3 College of Science and Technology, Bellevue University, Bellevue, NE 68005, USA; jkyndt@bellevue.edu

In the original publication [1], there was a mistake in the NRRL bacterial strain accession number. The numbers 5 and 1 were reversed. Strain C11^T^ is accessible though NRRL B-65718, not NRRL B-61758.

To comply with Rules 27 and 30 of the ICNP, and validate this new bacterial species, the entire corrected Abstract and Conclusions are as follows:

## Corrected Abstract

**Abstract:** A polyphasic taxonomic study was carried out on the rod-shaped, orange-pigmented strain C11^T^, isolated from gold mine tailings. Sequencing of the 16S rRNA gene showed a relatedness to *Brevundimonas*, with a 98.4% and 98.2% similarity to *Brevundimonas bacteroides* and *Brevundimonas variabilis*, respectively. The average nucleotide identity and a digital DNA–DNA hybridization with the closest phylogenetic neighbor of strain C11^T^ indicate distinction at the species level, further confirmed by the differences in physiology. C_18:1_ ω7c is the dominant cellular fatty acid. Its DNA G + C content is 68.3 mol %. Its predominant ubiquinone is Q-10; 1,2-Di-O-acyl-3-O-α-D-glucopyranuronosyl glycerol, phosphatidylglycerol, 1,2-di-O-acyl-3-O-α-D-glucopyranosyl glycerol, and 1,2-di-O-acyl-3-O-[D-glucopyranosyl-(1→4)-α-D-glucopyranuronosyl] glycerol are its major polar lipid constituents. This bacterium produces bacteriochlorophyll *a* and tolerates high concentrations of (μg/mL) the following: tellurium (>1500), selenium (1000 to >5000), and vanadium (>5000) oxyanions. The data support the inclusion of the strain C11^T^ into the genus *Brevundimonas* as a new species with the proposed name *Brevundimonas aurifodinae* sp. nov. (C11^T^ = NRRL B-65718^T^; =DSM 118059^T^).

## Corrected Section 4.1

*Brevundimonas aurifodinae* (au.ri.fo.di’nae. L. neut. n. aurum, gold; L. fem. n. fodina, mine; N.L. gen. n. aurifodinae, indicates discovery at a gold mine) is Gram-negative, non-motile, non-spore forming, and obligately aerobic. Circular (1–2 mm), raised, orange colonies with entire margins and a mucoid consistency formed on the *Caulobacter* medium plates after 72 h. The cells are rod-shaped, 1.5–2.0 μm in length and 0.8–1.0 μm in width, non-prosthecate, and catalase- and oxidase-positive. Growth occurs in the following conditions (optimum): between 5 and 40 °C (30 °C), from a pH of 6.0 to 10.5 (8.0), and up to 2.0% NaCl (0%). The carbon sources utilized include the following: Gluconate, dextrin, D-cellobiose, gentibiose, sucrose, D-turanose, stachyose, α-D-lactose, D-melibiose, N-acetyl-D-glucosamine, N-acetyl-beta-D-mannosamine, N-acetyl-D-galactosamine, N-acetyl-muraminic acid, α-D-glucose, D-mannose, D-galactose, 3-methyl-glucose, D-fucose, L-fucose, L-rhamnose, D-mannitol, D-arabitol, myo-inositol, D-glucose-6-phosphate, D-fructose-6-phosphate, D-aspartic acid, glycyl-L-proline, L-alanine, L-aspartic acid, L-glutamic acid, L-serine, D-galacuronic acid, L-galacturonic acid, D-gluconic acid, D-glucuronic acid, glucuronamide, quinic acid, D-saccharic acid, D-lactic acid methyl ester, citric acid, α-keto-glutaric acid, D-malic acid, L-malic acid, bromosuccinic acid, α-hydroxybutyric acid, β-hydroxy-D, L-butyric acid, α-keto-butyric acid, acetoacetic acid, acetic acid, casamino acids, yeast extract, and bactopeptone. Alternatively, capric acid, adipic acid, phenylacetic acid, D-maltose, D-trehalose, D-raffinose, β-methyl-D-glucoside, D-salicin, D-fructose, inosine, D-sorbitol, glycerol, D-serine, L-arginine, L-histidine, L-pyroglutamic acid, pectin, mucic acid, p-hydroxy-phenylacetic acid, methyl pyruvate, L-lactic acid, ɣ-amino-butyric acid, propionic acid, ethanol, methanol, and formic acid were not used. Can grow without vitamin supplements. Alkaline phosphatase, esterase (C4), esterase lipase (C8), leucine arylamidase, valine arylamidase, trypsin, α-chymotrypsin, acid phosphatase, naphthol-AS-bi-phosphohydrolase, α-glucosidase, and amylase activities were present, while arginine dihydrolase, lipase, cystine arylamidase, α-galactosidase, β-galactosidase, β-glucuronidase, β-glucosidase, N-acetyl-β-glucosaminidase, α-mannosidase, α-fucosidase, urease, and nitrate reductase were not. Esculin and gelatin were hydrolyzed. The indole, methyl red, and Voges–Proskauer tests were negative. The primary fatty acid was C_18:1_ ω7c. Ubiquinone Q-10 was the dominant isoprenoid quinone. MGDOx, PG, MGD, and DGL were the major polar lipids. It produces bacteriochlorophyll *a* and is resistant to high levels of (μg/mL) the following: tellurite (>1500), tellurate (>1500), selenite (1000), selenate (>5000), metavanadate (>5000), and orthovanadate (>5000). It can reduce tellurite to elemental tellurium. The DNA G + C content is 68.3 mol %.

The type strain C11^T^ (=NRRL B-65718^T^ = DSM 118059^T^) was isolated from gold mine tailings at Nopiming Provincial Park, in Manitoba, Canada. The strain C11^T^ ribosomal 16S rRNA gene sequence is available under the GenBank accession number: PP885399. This Whole Genome Shotgun project has been deposited at the DDBJ/ENA/GenBank under the accession JBEGDD000000000, which was used in this study.

The authors state that the scientific conclusions are unaffected. This correction was approved by the Academic Editor. The original publication has also been updated.
